# Factors Affecting the Duration of Hospitalization in Urology and Nephrology Patients in the Intensive Care Unit

**DOI:** 10.7759/cureus.67236

**Published:** 2024-08-19

**Authors:** Maheen Afzal, Mawra Noor, Mohammad Talal Afzal, Omar Yaseen

**Affiliations:** 1 Medicine, Jinnah Hospital, Allama Iqbal Medical College, Lahore, PAK; 2 Emergency Medicine, United Lincolnshire Hospitals NHS Trust, Lincolnshire, GBR; 3 Otorhinolaryngology, Norfolk and Norwich University Hospital, Norwich, GBR; 4 Medicine, Nottingham University Hospitals NHS Trust, Nottingham, GBR

**Keywords:** critical patients, urology, nephrology, length of stay in icu, medical intensive care unit (micu)

## Abstract

Introduction

Prolonged stays in the intensive care unit (ICU) are known to increase the risk of adverse outcomes following severe conditions. This study focuses on the factors affecting ICU length of stay, particularly in Urology and Nephrology patients who require intensive monitoring and specialized care, and their impact on patient outcomes.

Methods

A descriptive cross-sectional study was conducted with a sample size of 45 patients. Data was collected using a validated closed-ended questionnaire and analyzed with IBM SPSS Statistics for Windows, Version 23.0 (Released 2015; IBM Corp., Armonk, New York, United States).

Results

The study found positive associations between prolonged ICU stay and several factors, including elevated total bilirubin levels, deranged creatinine, urea, sodium, and calcium levels, as well as abnormal C-reactive protein levels. Higher bilirubin levels and positive blood and culture results were linked to extended ICU stays. Additionally, the use of contrast CT and MRI was associated with longer ICU durations. Conversely, broad-spectrum antibiotics showed a negative association with ICU stay length, while transfusion of blood products was positively associated with longer stays.

Conclusion

Understanding these factors can help improve the management of ICU patients and enhance outcomes in future cases.

## Introduction

An intensive care unit (ICU) offers a comprehensive range of monitoring and life-sustaining technologies. It serves as a central unit for treating severely ill patients in the hospital and can contribute to advancing the field of intensive care through research and educational efforts. An ICU has the capacity to deliver oxygen, conduct noninvasive monitoring, and provide more comprehensive nursing care compared to a regular ward. Additionally, for a particular duration, the ICU is equipped to furnish invasive monitoring and fundamental life support [1]. The care administered in ICUs demands significant financial resources and a substantial amount of manpower. Additionally, due to the scarcity of available ICU beds, the majority of ICUs operate at or close to maximum capacity [2].

Patients suffering from urological and nephrological disorders admitted to the ICU are managed with meticulous care provided by highly trained medical staff. These patients will receive comprehensive care, which includes ongoing monitoring of vital signs, managing hemodynamics, ensuring pain relief, monitoring renal function, providing respiratory support, and implementing infection control measures. Consequently, the period of stay in the ICU marks a significant milestone in the hospitalization journey for these patients [3].

Patients with life-threatening nephrological and urological conditions are admitted to the ICU to ensure that life-saving interventions are carried out with precision. Extended stays in the ICU lead to increased expenses and have a significant impact on both patients and their caregivers. Moreover, prolonged ICU stays have an adverse effect on their mortality rates. To enhance the quality of medical care, the main objective in intensive care is to minimize the duration of stay and lower the overall expenses of these patients [4].

There are various clinical and non-clinical factors that are associated with the duration of hospitalization in patients suffering from nephrology and urology disorders. Some of the clinical factors that can influence the duration of stay of these patients in the ICU can be broadly classified into biochemical investigations such as renal function tests, liver function tests, complete blood count, different types of cultures, inflammatory markers, imagining investigations with and without contrast, administration of antibiotics, and administration of blood products [4]. Some of the nonclinical factors are satisfaction regarding the psychological and social support of these patients [5,6].

One of the primary objectives of our study was to identify and categorize the factors associated with prolonged stays in the ICU for patients with renal conditions. Secondly, there is a noticeable lack of research concerning the factors influencing ICU stay duration specifically for nephrology and urology patients in our region. Consequently, our aim was to identify the key elements linked to extended ICU stays and provide statistical evidence that can aid healthcare professionals in making informed decisions to enhance the quality of care in our country. Lastly, this study sought to explore the relationship between various factors affecting ICU stay length and to offer recommendations on how these factors can be integrated into decision-making processes to improve resource allocation within the ICU.

## Materials and methods

Data for this study were collected from Begum Akhtar Rukhsana Memorial Trust Safari Hospital, Rawalpindi, Pakistan, from November 2022 to July 2023. Informed voluntary consent was obtained from all participants. The study was conducted with no anticipated risk or harm to participants, and it was approved by the Begum Akhtar Rukhsana Memorial Trust Safari Hospital Ethics Committee (approval number: BIHS/Adm/CME/24/12). Data were collected from patients' medical records and caregivers on the last day of ICU admission, following the acquisition of informed consent.

A descriptive cross-sectional dataset was compiled with a sample size of 45, calculated using the WHO sample size calculator with a 95% confidence interval. Non-probability purposive sampling was used for participant selection.

Inclusion and exclusion criteria

Participants were required to have nephrology or urology-related health issues necessitating ICU admission, such as acute kidney injury, chronic kidney disease, renal failure, urosepsis, urinary obstruction, or urological cancers. Patients had to be 18 years or older at the time of ICU admission and have primary pathologies directly related to nephrology or urology. Patients were excluded if they did not provide consent to participate, were under 18 years of age, or were admitted to the ICU for conditions unrelated to nephrology or urology. This ensured that the study focused specifically on relevant nephrology and urology cases and that all participants were capable of providing informed consent.

Data collection tool

The data collection tool employed was a validated closed-ended questionnaire, divided into two sections: Part A, which addressed the demographic details of the participants, and Part B, which covered aspects such as ICU stay duration, hospitalization outcomes, renal and liver function tests, complete blood counts, cultures, administration of broad-spectrum antibiotics and blood product transfusions, inflammatory markers, and imaging modalities (see Appendices).

Data analysis

Data analysis was performed using IBM SPSS Statistics for Windows, Version 23.0 (Released 2015; IBM Corp., Armonk, New York, United States), with a significance threshold set at a p-value of < 0.05. Quantitative data were expressed as mean and standard deviation (SD), while qualitative data were presented as frequencies and percentages. Investigations were categorized into normal and deranged values. The correlation between continuous and categorical variables was assessed using point-biserial correlation, and statistical significance between independent groups was determined using the independent sample t-test.

## Results

The study included 45 participants. Of these, 26 (57.8%) were male and 19 (42.2%) were female. The mean age was 56.91 ± 18.1 years, with a median age of 56 years. The average length of stay in the intensive care unit was 5.33 ± 4.29 days, with a median of four days and a range of 1-22 days. The reasons for admission were as follows: 26 (59%) had multiple diseases (chronic kidney disease, diabetes mellitus, hypertension), seven (15.6%) had diabetes mellitus, three (6.7%) had hypertension, one (1%) had respiratory disease, one (2.2%) had cardiovascular diseases, and seven (15.5%) had urogenital diseases. Outcomes for the 45 patients were as follows: four patients (8.9%) showed improvement, four patients (8.9%) were discharged, 13 patients (28.9%) died, and 24 patients (53.3%) were transferred to other wards after management.

Analysis of the collected data revealed a negative association between abnormal serum alanine transaminase (ALT), aspartate aminotransferase (AST), and albumin levels and the duration of stay in the ICU. Conversely, there was a positive association between total bilirubin levels and ICU stay duration, indicating that higher bilirubin levels were associated with a longer ICU stay (Table 1).

**Table 1:** Correlation between duration of stay and liver function tests ALT: alanine transaminase; AST: aspartate aminotransferase

S.no.	Liver Function Tests	Pearson Correlation	p-value
1.	Serum ALT levels in U/L	-0.031	0.841
2.	Serum AST levels in U/L	-0.098	0.522
3.	Total bilirubin levels in mg/dl	0.204	0.180
4.	Serum Albumin levels in g/dl	-0.047	0.757

Analysis of the data revealed a negative association between serum potassium levels and the duration of ICU stay. Additionally, there was a positive association between elevated creatinine, urea, sodium, and calcium levels and the length of ICU stay (Table 2).

**Table 2:** Correlation between duration of stay and renal function tests

S no.	Renal Function Tests	Pearson Correlation	p-value
1.	Serum Creatinine levels	0.021	0.891
2.	Blood Urea levels	0.108	0.481
3.	Serum Sodium levels	0.024	0.875
4.	Serum Potassium levels	-0.091	0.550
5.	Serum Calcium levels	0.293	0.050

Analysis of the data showed that only positive blood and other sample cultures were associated with an increased duration of ICU stay. In contrast, positive throat, urine, and nasal cultures were linked to a shorter ICU stay (Table 3).

**Table 3:** Correlation between duration of stay and cultures and sensitivity tests C/S: culture and sensitivity

S. no	Cultures and sensitivity	Pearson Correlation	p-value
1.	Blood C/S	0.105	0.493
2.	Throat C/S	-0.033	0.828
3.	Urine C/S	-0.133	0.383
4.	Nasal C/S	-0.067	0.664
5.	Others C/S	0.008	0.958

Analysis of the data revealed a negative association between abnormal platelet levels and ICU stay duration. Conversely, deranged blood hemoglobin levels and white blood cell (WBC) counts were associated with a longer ICU stay (Table 4).

**Table 4:** Correlation between duration of stay and blood tests WBC: white blood cell

S. no.	Blood Tests	Pearson Correlation	p-value
1.	Hemoglobin	0.031	0.841
2.	Platelet	-0.166	0.277
3.	WBC	0.204	0.180

There was a positive association between abnormal C-reactive protein levels and the duration of ICU stay, while deranged procalcitonin levels were negatively associated with ICU stay duration (Table 5).

**Table 5:** Correlation between duration of stay and Inflammatory markers

S. no.	Inflammatory Markers	Pearson Correlation	p-value
1.	C-reactive protein	0.064	0.676
2.	Procalcitonin	-0.078	0.612

Analysis of the data revealed a negative association between the administration of broad-spectrum antibiotics and the duration of ICU stay, while transfusion of blood products was positively associated with a longer ICU stay (Table 6).

**Table 6:** Correlation between duration of stay and medical intervention

S. no.	Medical intervention	Pearson Correlation	p-value
1.	Administration of broad-spectrum antibiotics	-0.152	0.319
2.	Transfusion of blood products	0.045	0.774

Analysis of the data showed that the use of contrast CT and MRI was associated with a longer ICU stay (Table 7).

**Table 7:** Correlation between duration of stay and imaging studies

S. no.	Imaging Studies	Pearson Correlation	p-value
1.	Plain CT and MRI	-0.211	0.164
2.	Contrast CT and MRI	0.109	0.475

The data indicate that psychological support reduced the duration of the ICU stay, while social support provision was associated with a longer stay (Table 8).

**Table 8:** Correlation between duration of stay and support provision

S. no.	Support provision	Pearson Correlation	p-value
1.	Psychological satisfaction	-0.081	0.596
2.	Social support	0.160	0.293

## Discussion

Our study aimed to investigate the correlation between ICU duration and the influencing factors, but there was a study conducted on critically ill patients related to cardiovascular diseases highlighting that extended stays in the ICU are associated with higher mortality rates for patients [7]. 

Our study indicates that abnormal bilirubin levels were linked to prolonged stays in the ICU. The Simplified Acute Physiology Score used to predict mortality in critically ill patients takes into account total bilirubin levels to estimate the probability of mortality on the first day of hospital admission since there is an indirect link between abnormal bilirubin levels and prolonged ICU stays in cardiovascular patients [7]. Similarly, a study by Zheng et al. states that a higher serum total bilirubin on ICU admission was independently associated with mortality in acute respiratory distress syndrome (ARDS) patients [8].

In our study, we observed a positive correlation between abnormal levels of creatinine, urea, sodium, and calcium and the length of stay in the ICU of urology and nephrology patients. Toptas et al. also emphasized, in their study, that as values of urea, creatinine, and sodium rise, so does the duration of stay [4]. This underscores the importance for physicians to closely monitor kidney function and ensure proper hydration. A study in 2012 by Mousavi et al. states that mortality of ICU patients is linked, in greater part, to organ dysfunction, but the severity of serum sodium and potassium disturbances remains a significant predictor of mortality [9]. Thus, correcting electrolyte disturbances in ICU patients is important.

In a study by Schneider et al., the presence of acute kidney injury during the pediatric intensive care unit (PICU) stay, or any deterioration in serum creatinine as stratified RIFLE (Risk, Injury, Failure, Loss of kidney function, and End-stage kidney disease) scores, was associated with an extended length of stay in the PICU [10]. 

Another study by Jeganathan et al. emphasized that comprehending the frequency of microbiological positivity could influence the use of diagnostic methods and biomarkers, prompting additional research to assess their efficacy in septic patients with unidentified infection sites or negative microbiology [11]. In our study, upon scrutinizing the gathered data, we observed that only blood and other sample cultures displayed positivity, leading to an extended ICU stay and alternating the prognosis. Conversely, positivity in the throat, urine, and nasal cultures of urology and nephrology patients correlated with a reduced duration of ICU stays. 

There was a relationship between iron-restricted anemia and sepsis in a study by Loftus et al. leading to a worse prognosis in critically ill patients, and it was noted that a prolonged elevation of inflammatory cytokines was linked to iron-restricted anemia in critically ill septic patients [12]. This occurred even in the absence of systemic iron deficiency and was independent of endogenous erythropoietin levels. However, there was no association between anemia, sepsis, and length of stay in the ICU patients. In our study, our data analysis further revealed that abnormal blood hemoglobin levels and WBC count led to an extended duration of stay of urology and nephrology patients in the ICU. 

A study conducted on psychological problems following ICU treatment in the United Kingdom highlights the importance of providing psychological support to survivors of critical illness. It states that assessment and treatment of psychological distress in discharged ICU patients is needed because the prevalence of post-traumatic stress, depression, and anxiety conditions appears to be high [13]. This support can not only improve their perceived health and reduce anxiety but also potentially lead to shorter hospital stays, which can be beneficial for both the patients and the healthcare system as a whole. Early intra-ICU intervention by clinical psychologists may help critically ill trauma patients recover from the stressful experience [14].

Timely administration of antibiotics is crucial for patients with severe sepsis and septic shock. Administering antibiotics within three hours of arrival has been associated with improved outcomes, including higher survival rates, organ function recovery, and shorter hospital stays [15]. Another study has demonstrated the negative impact of inadequate empiric antimicrobial therapy (EAMT) on the outcomes of sepsis patients. It states that inadequate EAMT was significantly associated with poor clinical outcomes in these patients including increased mortality and longer length of hospital stay [16]. Furthermore, our study suggests an interesting finding regarding the negative association between the administration of broad-spectrum antibiotics and ICU stay duration. This might imply that prompt and targeted antibiotic therapy could lead to more efficient treatment and potentially shorter ICU stays. 

Contrast-induced nephropathy is a significant concern in healthcare settings, as it can lead to acute kidney insufficiency and is associated with adverse outcomes like increased hospital mortality, prolonged length of stay, and higher healthcare costs [17]. The finding of the current study that using contrast CT and MRI resulted in more days of stay in the ICU is noteworthy. This finding underscores the importance of careful consideration when choosing imaging techniques for patients, particularly those at risk for contrast-induced nephropathy.

Limitations

Ours was a descriptive cross-sectional study, making the results susceptible to prevalence/incidence bias, which limits their generalizability. Additionally, the short duration of the study and the small sample size further hinder the ability to generalize the findings. To fully understand the relationship between ICU stay duration and various factors, multiple longitudinal studies are needed.

## Conclusions

Factors affecting ICU stay length in Urology and Nephrology patients who required close monitoring and specialized medical staff were investigated. The study found a positive association between prolonged ICU stays and elevated levels of total bilirubin, creatinine, urea, sodium, and calcium, as well as abnormal C-reactive protein levels. Higher bilirubin levels correlated with longer ICU stays. Additionally, positive blood and other sample cultures were linked to extended ICU durations. Data analysis revealed that the use of contrast CT and MRI also resulted in longer ICU stays. Conversely, broad-spectrum antibiotics were negatively associated with ICU duration, while transfusion of blood products was positively associated with it. Understanding these factors could help reduce ICU stay lengths, potentially impacting mortality rates and improving the quality of patient care.

## Appendices

Data collection tool

**Table 9:** Data collection form

Field	Data
Date	
ID	
Consent Given	Yes / No
Age	______ Years
Gender	_______
Income	_______ PKR
Duration of stay	_______ Days
Outcome of hospitalization	Improved / Discharge with follow up / LAMA / Refer to other specialty / Still under treatment in ICU / Exitus
Association with any other disease	Diabetes Mellitus / Hypertension / Cerebrovascular disease / GIT diseases / respiratory diseases / Others
Renal Function Tests	Urea _________ Creatinine__________
Electrolytes	Na+____________ K+_____________ Ca2+___________
Blood complete picture	Hgb________ Platelets_________ WBCS___________ RBCs____________
Inflammatory Markers	CRP______________ PCT______________
Liver function tests:	ALT__________ AST__________ Total bilirubin __________ Albumin ___________
Culture reports	Blood C/S__________ Throat C/S__________ Urine C/S___________ Nasal C/S___________ Other C/S___________
Broad spectrum use of antibiotics	
Blood products transfusions	
Plain CT and MRI	Yes / No
Contrast CT and MRI	Yes / No
Psychological support during stay in ICU	Satisfactory / unsatisfactory
Social support during stay in ICU	Satisfactory / unsatisfactory
